# *In Vivo* Occupancy of Mitochondrial Single-Stranded DNA Binding Protein Supports the Strand Displacement Mode of DNA Replication

**DOI:** 10.1371/journal.pgen.1004832

**Published:** 2014-12-04

**Authors:** Javier Miralles Fusté, Yonghong Shi, Sjoerd Wanrooij, Xuefeng Zhu, Elisabeth Jemt, Örjan Persson, Nasim Sabouri, Claes M. Gustafsson, Maria Falkenberg

**Affiliations:** 1 Department of Medical Biochemistry and Cell Biology, University of Gothenburg, Gothenburg, Sweden; 2 Department of Medical Biochemistry and Biophysics, Umeå University, Umeå, Sweden; Max Planck Institute for Biology of Ageing, Germany

## Abstract

Mitochondrial DNA (mtDNA) encodes for proteins required for oxidative phosphorylation, and mutations affecting the genome have been linked to a number of diseases as well as the natural ageing process in mammals. Human mtDNA is replicated by a molecular machinery that is distinct from the nuclear replisome, but there is still no consensus on the exact mode of mtDNA replication. We here demonstrate that the mitochondrial single-stranded DNA binding protein (mtSSB) directs origin specific initiation of mtDNA replication. MtSSB covers the parental heavy strand, which is displaced during mtDNA replication. MtSSB blocks primer synthesis on the displaced strand and restricts initiation of light-strand mtDNA synthesis to the specific origin of light-strand DNA synthesis (OriL). The *in vivo* occupancy profile of mtSSB displays a distinct pattern, with the highest levels of mtSSB close to the mitochondrial control region and with a gradual decline towards OriL. The pattern correlates with the replication products expected for the strand displacement mode of mtDNA synthesis, lending strong *in vivo* support for this debated model for mitochondrial DNA replication.

## Author Summary

Mitochondria are cytoplasmatic organelles that produce most of the adenosine triphosphate (ATP) used by the cell as a source of chemical energy. A subset of proteins required for ATP production is encoded by a distinct mitochondrial DNA genome (mtDNA). Proper maintenance of mtDNA is essential, since mutations or depletion of this circular molecule may lead to a number of different diseases and also contribute to normal ageing. We are interested in the molecular mechanisms that ensure correct replication and propagation of mtDNA. Even if many of the responsible enzymes have been identified, there is still a debate within our scientific field regarding the exact mode of mtDNA replication. We have here used a combination of *in vitro* biochemistry and *in vivo* protein-DNA interaction characterization to address this question. Our findings demonstrate that the mitochondrial single-stranded DNA-binding protein (mtSSB) restricts initiation of mtDNA replication to a specific origin of replication. By characterizing how mtSSB interacts with the two strands of mtDNA *in vivo*, we are able to directly demonstrate the relevance of one proposed mode of mitochondrial DNA replication and at the same time seriously question the validity of other, alternative modes that have been proposed over the years.

## Introduction

In their inner membrane, mitochondria harbor the oxidative phosphorylation (OXPHOS) system, which generates ATP needed to drive energetically unfavorable cellular reactions. Most OXPHOS components are encoded in the nuclear genome, but genes for 13 essential subunits are encoded by a separate mitochondrial DNA genome (mtDNA). Mutations, deletions and depletion of mtDNA result in defective energy production, which in turn causes a wide variety of disease symptoms [1].

Human mtDNA is a relatively small (∼16,6 Kb) circular double-stranded molecule that is organized into nucleoprotein complexes, denoted nucleoids [2], [3], [4]. The two strands of the mitochondrial genome are known as the heavy strand (H-strand) and light strand (L-strand), owing to a strand bias in guanine and thymine base content. The genome is replicated by a molecular machinery that is distinct from the nuclear replication apparatus. The core components of this machinery are related to their phage T7 counterparts, including the catalytic subunit of DNA polymerase γ (POLγA), the DNA helicase TWINKLE, and the mitochondrial RNA polymerase (POLRMT), which synthesizes RNA primers during initiation of DNA synthesis [5], [6]. RNase H1 is also required for mtDNA maintenance and likely plays a role for primer removal during mtDNA maturation [7].

The mitochondrial single-stranded DNA binding (mtSSB) protein does not have a phage ancestry, but instead resembles *Escherichia coli* SSB [8]. MtSSB binds to single-stranded DNA (ssDNA) as a tetramer composed of four 16 kDa subunits [9]. The protein stimulates synthesis of mtDNA [10] by facilitating POLγ primer recognition [11] and enhancing POLγ processivity [12]. MtSSB also stimulates the dsDNA unwinding activity of TWINKLE [13].

Currently there is no consensus regarding the mechanism by which mammalian mtDNA is replicated. Early work reported that mtDNA replication occurs through a strand-displacement mode (SDM) [14]. According to this model, mtDNA synthesis is continuous on both strands. DNA synthesis is first initiated at the origin of heavy-strand DNA replication (OriH) and nascent H-strand DNA synthesis continues to displace the parental H-strand. During the first stage of DNA synthesis, there is no simultaneous light-strand (L-strand) DNA synthesis. When two-thirds of the H-strand has been synthesized, the replication machinery reaches the origin of light-strand DNA replication (OriL). At this point, the H-strand of OriL is exposed in its single-stranded conformation and it folds into a stem-loop structure. The folded origin is recognized by POLRMT, which initiates primer synthesis from a short T-stretch in the single-stranded loop region of the activated origin. After about 25 nts, POLRMT is replaced by POLγ at the 3′-end of the RNA primer and lagging-strand DNA synthesis is initiated. After initiation, H- and L-strand DNA synthesis proceeds continuously until each strand is completely replicated and two daughter molecules are formed. SDM replication intermediates have been identified and characterized by various biochemical techniques (for a detailed review, please see [15]). In recent years, key steps in the process have also been reconstituted *in vitro* with purified proteins [6], [16], [17]. The SDM is also supported by evolutionary analysis that revealed an asymmetric skew in bases frequencies at the third codon position in mitogenomes. This mutational gradient correlates with the time the H-strand would be in its more mutagenic single-stranded conformation according to SDM [18].

A number of studies have questioned the *in vivo* relevance of SDM and suggested the existence of two other, alternative models for mtDNA replication. The so-called ribonucleotide incorporation throughout the lagging strand (RITOLS) model is primarily based on data obtained by neutral two-dimensional agarose gel electrophoresis (2D-AGE) [19], [20], [21]. The authors of these studies concluded that the mtDNA replication intermediates observed in 2D-AGE cannot be explained by SDM replication and they have also criticized the SDM for limited *in vivo* evidence [22]. RITOLS and SDM replication are very similar, with the exception of one striking difference, the requirement of mtSSB. According to the SDM, the displaced H-strand is single-stranded, and therefore coated with mtSSB, which is subsequently removed during L-strand synthesis. In contrast, the RITOLS replication model states that RNA covers the displaced lagging-strand during replication [20]. According to the RITOLS model, the RNA intermediates associated with mtDNA replication are processed transcripts (including tRNAs), which are successively treaded onto the exposed lagging-strand template after passage of the replication fork [21]. A weakness of the RITOLS model is that the enzymes required for this process, i.e. hybridization of RNA intermediates, have not been defined.

Whereas both the SDM and RITOLS models imply strand asynchronous mtDNA replication, there is also a third model, which suggests strand coupled (SC) replication of the mtDNA [19]. The SC model is based on the identification of fully duplex DNA replication intermediates in 2D-AGE analysis, which were seen as evidence for coupled leading and lagging strand DNA replication. These products are generally only a minor fraction of all replication intermediates, but they can be detected under specific experimental conditions [21].

We reasoned that a detailed characterization of mtSSB occupancy and its function during mtDNA replication could help to elucidate the mechanism for mtDNA replication. We here demonstrate that mtSSB exclusively covers the H-strand during mtDNA replication and helps to direct primer synthesis to OriL. Our findings reveal the molecular basis for origin specific initiation of light-strand mtDNA synthesis and provide strong experimental support for the *in vivo* relevance of the SDM replication model.

## Results

### MtSSB directs OriL specificity

**Figure 1:**
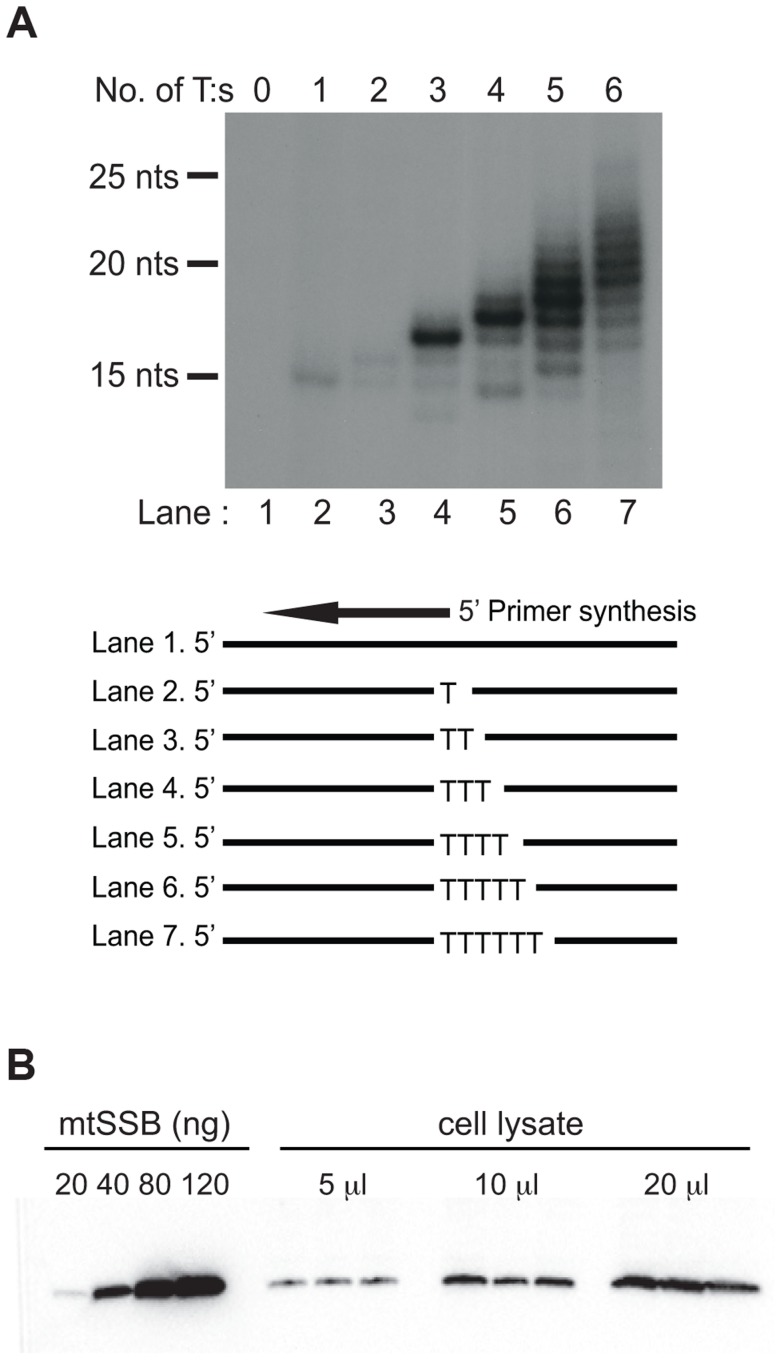
POLRMT can initiate primer synthesis from a single dT and mtSSB is abundant *in vivo*. (A). POLRMT can initiate primer synthesis from a linear template containing one or more dT (lanes 2 to 7). Deletion of the poly-dT stretch abolishes primer synthesis (lane 1). (B) Representative quantitative Western blot measurement of endogenous mtSSB protein in human Hela cells. Protein extracts (5, 10 or 20 µl) were loaded from a determined number of cells. Purified recombinant mtSSB was used to create standard curve with known protein concentrations.

Both the SDM and RITOLS models imply that the parental H-strand will be single-stranded for a long period of time during mtDNA replication. This conformation presents a problem, since POLRMT has limited sequence requirements and it may readily initiate primer synthesis outside the OriL region. POLRMT uses ATP as the initiating nucleotide and only requires a single T (thymine base) in the template strand to start primer synthesis (Fig. 1A, lane 2). The reaction is further stimulated on templates containing a poly-T stretch (Fig. 1A, compare lane 2–7). Given the limited sequence requirement of POLRMT on ssDNA, it is essential to protect the displaced H-strand in order to prevent unrestricted initiation of L-strand DNA synthesis.

We decided to investigate if mtSSB (as expected for SDM) or processed transcripts (as predicted for RITOLS) was responsible for protecting the parental H-strand. To this end, we first analyzed the *in vivo* levels of mtSSB to establish if the protein was sufficiently abundant to coat the parental H-strand during mtDNA synthesis. We isolated mitochondria from HeLa cells and performed quantitative immunoblot analysis with antibodies against mtSSB. The protein concentration was determined by comparison with known amounts of recombinant mtSSB protein (Fig. 1B). In parallel, we determined mtDNA copy number by quantitative real-time PCR, which allowed us to calculate a ratio of about 2,100 molecules of mtSSB per mtDNA molecule (Table 1). Our values agreed nicely with previous estimates of 3,000 molecules of mtSSB per mtDNA molecule [23]. MtSSB binds to single-stranded DNA as a tetramer, corresponding to at least 500 mtSSB tetramers available per mtDNA molecule in the cell. Each tetramer of mtSSB has been reported to bind 59 nts [24], which implies that there is more than enough mtSSB to coat the entire parental H-strand during mtDNA replication.

**Table 1:** Quantification of mtSSB to mtDNA ratio in HeLa cells.

mtDNA molecules per cell	5.07±0.06 E+03
mtSSB molecules per cell	1.08±0.45 E+07
mtSSB per mtDNA	2.13±0.88 E+03

**Figure 2:**
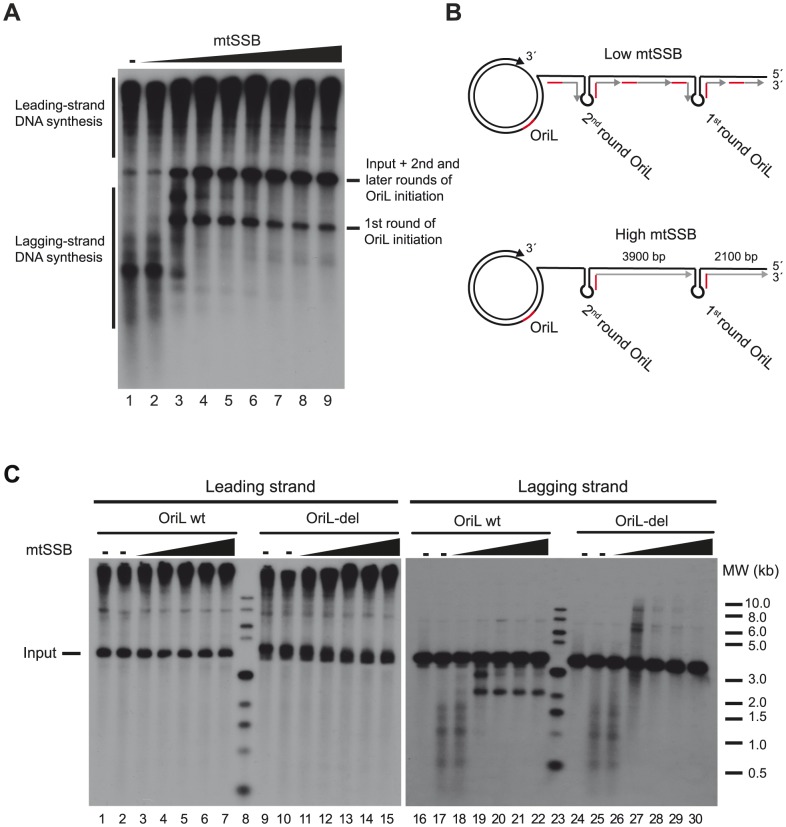
mtSSB governs OriL specificity. (**A**) *In vitro* rolling circle DNA replication reaction with increasing concentrations of mtSSB (0, 10, 100, 500 fmol, 1, 5, 10, 20 and 40 pmol) on the SK+OriL dsDNA template. DNA replication was performed in the presence of [α- 32P]-dCTP in order to label newly synthesized DNA as described previously [6]. The weak labeling of input template in lanes 1 and 2 is most likely due to POLγ idling on the free 3′-end, in the absence of active rolling circle DNA replication. (**B**) Schematic illustration explaining the replication products formed on lagging-strand DNA. At higher mtSSB levels, primers synthesis is restricted to OriL, but at lower levels primer synthesis can take place also at other sites. During the first round of DNA synthesis, the OriL-depending lagging-strand products have a length of about 2100 nts. In later rounds, the fragments will span the entire distance between two OriL sequences (about 3900 nts) and migrate with the same size as the input template. (**C**) Reactions were performed as in panel A, but replication products were analyzed by Southern blotting using strand-specific probes to detect leading- or lagging-strand DNA synthesis. For comparison, we used a mutant template (OriL-del) in which the OriL sequence had been deleted.

We next investigated if mtSSB's ability to prevent random primer synthesis [16] could direct OriL specific initiation of lagging-strand synthesis *in vitro*. To this end, we combined POLγ, the mitochondrial helicase TWINKLE and mtSSB, and studied DNA synthesis on a double stranded template with a preformed replication fork [25]. In the presence of POLRMT, which acts as a primase for initiation of light-strand DNA synthesis at OriL, the system can catalyze the formation of dsDNA products *in vitro* (Fig. 2 A and B lower panel) [6].

When mtSSB was omitted or added at low levels to the rolling circle replication assay, we observed a smear of shorter products suggesting that primer synthesis and initiation of lagging-strand DNA synthesis could take place at multiple sites on the DNA template. Under these conditions, it was difficult to observe OriL specific initiation events (Fig. 2A, lane 1–2 and Fig 2B, upper panel). Please note that we are using saturating levels of TWINKLE and POLγ, which explain why mtSSB only has a minor stimulatory effect on leading-strand DNA synthesis [25]. When we added increasing levels of mtSSB, we began to observe a specific product corresponding to the expected size for OriL-dependent lagging-strand initiation. At higher mtSSB concentrations the smear of shorter products completely disappeared and only the OriL-specific band remained intact (Fig 2A lane 7–9 and Fig. 2B lower panel). Our findings demonstrated that mtSSB restricts initiation of lagging-strand DNA synthesis *in vitro* to OriL.

To further verify our interpretations, we performed similar experiments, but analyzed the replication products with Southern blotting, using leading- and lagging-strand specific probes. As a negative control, we used a rolling-circle template lacking the OriL sequence (OriL del). We observed efficient leading-strand DNA synthesis with both the wt and OriL del template (Fig. 2C left panel). The leading-strand specific probes detected replication products of 3,900 nts (input template) and longer. Using the lagging-strand specific probes and in the absence of mtSSB, we detected multiple, shorter DNA synthesis products on both the wt and OriL del template (Fig 2C right panel lanes 17–18 and 25–26). When we increased the mtSSB concentrations, a product with a size corresponding to that expected for replication products initiated at OriL replaced the smear of shorter products produced with the wt template (Fig. 2C lane 19–22). The band was not observed when the OriL del was used as template (Fig. 2C lane 27–30) We could thus conclude that mtSSB restricts initiation of lagging-strand DNA synthesis to OriL in our reconstituted *in vitro* system.

### The OriL stem-loop structure prevents mtSSB binding

**Figure 3:**
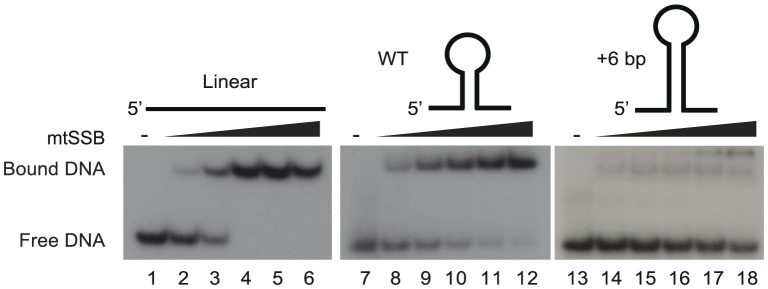
The stem-loop structure of OriL prevents mtSSB binding. MtSSB binding to ssDNA oligonucleotides was monitored by EMSA as described in experimental procedures. Increasing mtSSB concentrations (0, 5,10, 25, 50 100 fmol mtSSB calculated as a tetramer) were incubated for 15 minutes at room temperature with 25 fmol of [γ- 32P]-ATP 5′-end labeled DNA. As templates we used WT OriL, an OriL derivative lacking a stem or an OriL derivative with a 6 bp increase in stem length.

We have previously suggested that the stem-loop structure of activated OriL prevents mtSSB binding, thereby making the T-stretch in the single-stranded loop region available for initiation of primer synthesis by POLRMT (Figure S1 and [6]). To directly test this idea, we used gel shift analyses to monitor binding of mtSSB to OriL containing DNA substrates and mutant derivatives thereof (Fig. 3). MtSSB formed a complex and no free template was observed when mtSSB was added at a molar ratio of 1∶1 or higher relative to a single-stranded DNA oligonucleotide without any predicted secondary structure potential (Fig. 3, lanes 1–6). MtSSB binding to an OriL containing oligonucleotide was less efficient (Fig 3, lane 7–12). The effect was even more pronounced, when the stem region was stabilized by a 6-bp extension, which almost abolished mtSSB binding (Fig. 3, lane 13–18).

### MtSSB binds exclusively to the H-strand

**Figure 4:**
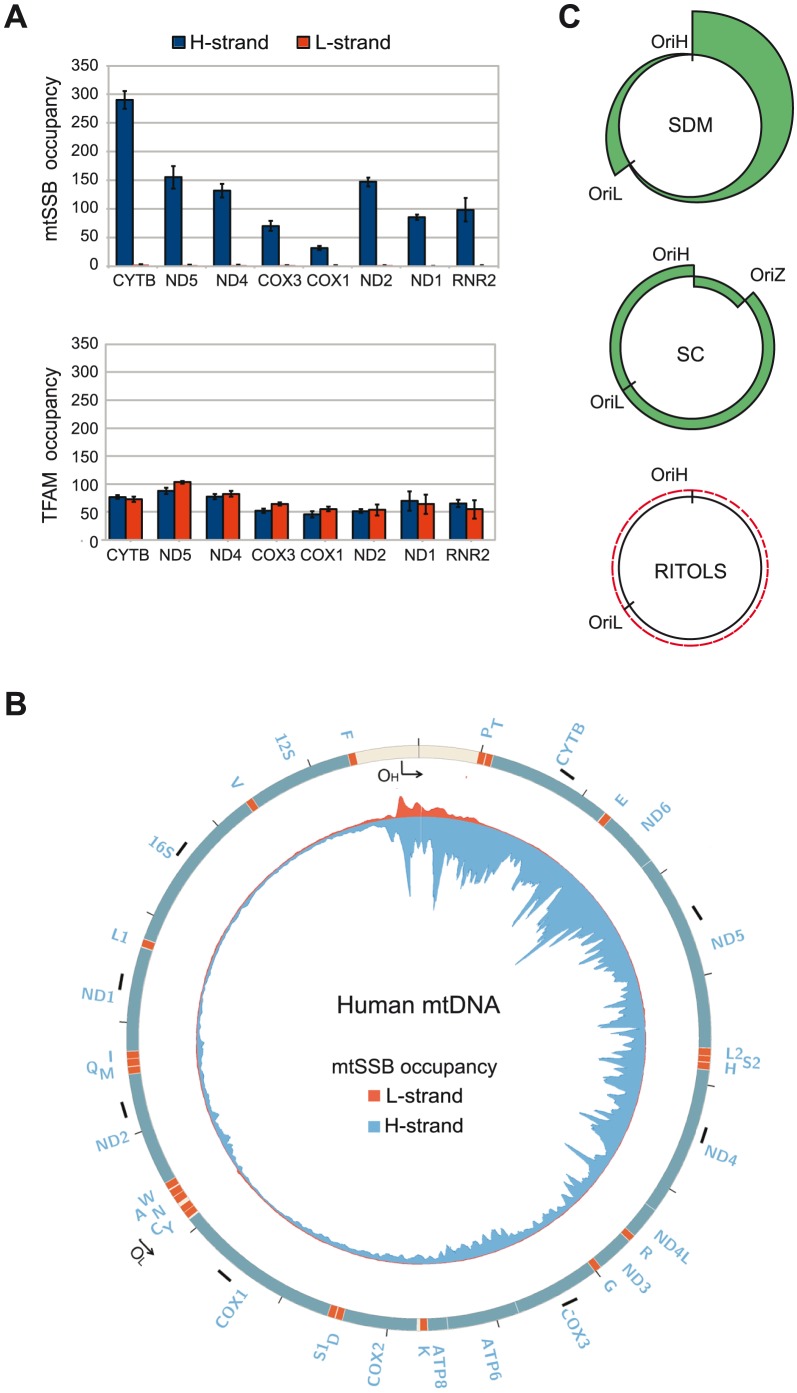
mtSSB *in vivo* occupancy reflects strand-displacement mtDNA replication. (**A**) Occupancy of mtSSB and TFAM analyzed by strand-specific qPCR amplification of ChIP samples. (**B**) Strand-specific ChIP-seq profile of mtSSB binding to mtDNA. The origins of replication are indicated. The short black bars indicate the location of the primers used for strand-specific qPCR. (**C**) Schematic illustration of expected occupancy of mtSSB accordingly to the different mtDNA replication models. SDM (Strand displacement mode), SC (Strand coupled mode), and RITOLS.

If correct, SDM would imply that mtSSB binds throughout the H-strand during mtDNA replication to block unspecific initiation of mtDNA synthesis at regions outside of OriL. To address if this was indeed the case *in vivo*, we investigated the binding pattern of mtSSB along the mtDNA genome using chromatin immunoprecipitation followed by real-time quantitative PCR. To this end, we designed primers that were specific for the two DNA strands, i.e. which could detect mtSSB binding to the H- or L-strand, respectively. Eight evenly distributed mtDNA regions were chosen for our analysis (Fig. 4B, short black bars and the primers used are listed in Table S1). First, strand-specific primers with distinct 5′ tag sequences were used to copy the H- or the L-strand. Next, the synthesized tagged-DNA was amplified by PCR with a primer corresponding to the tag sequence and a primer complementary to the target DNA. Our analysis revealed a striking strand bias in mtSSB binding (Fig. 4A). There were only back-ground levels of mtSSB binding to the L-strand, whereas we observed a strong signal from the H-strand. The findings were thus in nice agreement with our model based on *in vitro* experiments, which stated that mtSSB should protect the parental H-strand. Interestingly, mtSSB binding to the H-strand was not uniform, but displayed a distinct pattern. The highest mtSSB occupancy was observed in CYTB, just downstream of the D-loop region and after this point we observed a progressive decrease with a minima of mtSSB occupancy in COX1, just before OriL. A second smaller peak of mtSSB binding was observed just after OriL (in ND2), followed by a gradual decrease towards the control region (ND1 and RNR2) (Fig 4A, upper panel). The observed pattern therefore correlated with what would be expected for strand-displacement mtDNA replication (Fig 4C, upper panel). According to this model, the region just downstream of the control region (CYTB) would be present in its single-stranded conformation for a much longer time than the region next to OriL (COX1). Similarly, the region just downstream of OriL (ND2) would remain single-stranded longer than the regions just upstream of the control region (ND1 and RNR2). As a control, we also investigated the binding pattern of TFAM. A previous report has demonstrating that this dsDNA-binding protein is evenly distributed over the genome [26]. Using our strand-specific primers, we found that TFAM interacted with both the H- and L-strands to a similar extent (Compare Fig. 4A, upper and lower panel). We could thus conclude that the observed strand-bias was specific for mtSSB.

### Strand-specific mapping of mtSSB with next generation sequencing

In order to obtain a more detailed profile of mtSSB occupancy, we again employed strand-specific ChIP analysis, but analyzed the precipitated material by strand-specific next generation sequencing. We excluded sequences that mapped to the nuclear genome and reads without a perfect match to the mtDNA from further analysis. In total, we could map 17.4% of the mtSSB ChIP reads to the mitochondrial genome, which was about 85-fold higher than the mapped reads (0.2%) in the input samples, consistent with the fact that mtSSB is exclusively localized to the mitochondria. We could map 92.8% of the mtSSB ChIP reads to the H-strand (blue color), while 7.2% reads mapped to the L-strand (red color). Our sequencing data also revealed a distinct mtSSB-binding pattern, which correlated nicely with our strand specific ChIP-qPCR results. Outside the control region, mtSSB associated only with the H-strand and there was a negative gradient towards OriL. A second, smaller peak of mtSSB was observed just after OriL (Fig. 4B). These results support the idea that mtSSB stabilizes the H-strand during mtDNA replication and the pattern of occupancy is in agreement with the strand-displacement model for mtDNA replication. In the control region, we noticed binding to both the H- and L-strands. The reason for this pattern is not clear to us, but our findings could support a role of mtSSB in 7S DNA turnover, as previously suggested [23], [27].

### The mitochondrial replication intermediates support the SDM

**Figure 5:**
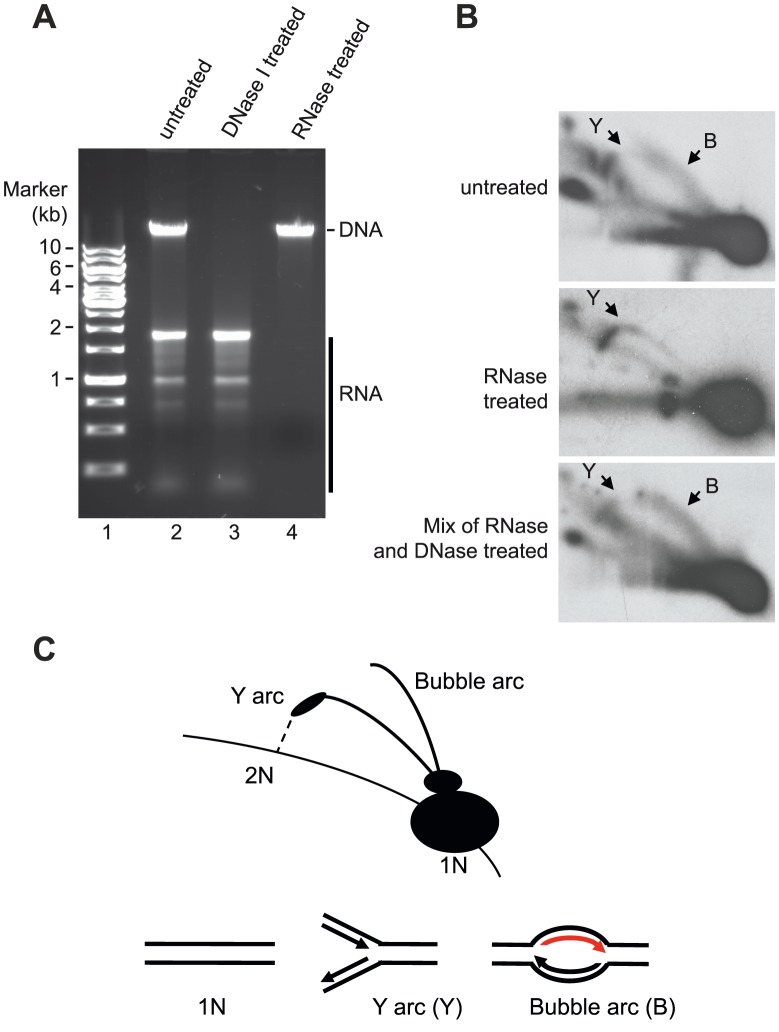
Neutral 2D-AGE analysis of mtDNA replication products. (**A**) Extracted DNA analyzed on 1% Agarose. (**B**) Purified DNA cut with HincII was analysed using 2D-AGE. A fragment (mtDNA 13636-1006 bp) spanning the OriH region was visualized with probe located in CYTB (14641-15590 bp). Upper panel; untreated DNA (containing both RNA and DNA), Middle panel; RNase A and RNase H treated DNA (containing only DNA); Lower panel, DNA treated with RNaseA and RNaseH remixed with the RNA still present after DNase I treatment. (**C**) Schematic illustration of how Y and bubble arcs are expected to run in 2D-AGE. The bubble arc observed here is dependent on RNA (indicated in red).

Our data demonstrated that mtSSB protects the single-stranded H-strand *in vivo*. This finding was at odds with the RITOLS model, in which the parental H-strand is covered with processed mitochondrial transcripts. The RITOLS model is primarily based on the observation of long RNA/DNA hybrids using neutral two-dimensional agarose gel electrophoresis (2D-AGE) [21], [28]. We decided to repeat the published 2D-AGE analysis of mtDNA replication products in an attempt to better understand these contradictory results. To this end we purified mitochondria from HeLa cells and isolated mtDNA following protocols previously used to identify RITOLS [29]. Agarose analysis revealed that the mtDNA preparations prepared this way contained large amounts RNA, which were much more abundant than the DNA in these samples (Fig. 5A, lanes 2 and 3). We confirmed that the observed RNA contained processed mitochondrial transcripts by Northern blotting of ND4, CYTB and COX3 transcripts (Figure S2). We next digested the isolated mtDNA with Hinc II and analyzed the replication products by 2D-AGE analysis (Fig. 5A, lane 2). For comparison, we also analysed samples in which we had removed RNA by treatment with RNase A and RNase H (Fig 5A, lane 4). For detection of replication intermediates, we performed Southern blotting using a radioactively labeled probe, which detected a Hinc II fragment spanning position 13,636 to 1,006 in mtDNA.

When we analyzed the mtDNA preparations containing RNA, we detected two prominent structures, which also have been described in previous reports; an Y arc and a bubble arc. The Y-arc is unrelated to the RITOLS model [19], [30] and how this structure may be formed will be addressed in the discussion (see below). Bubble arcs are typically seen during theta type DNA replication and in the case of the RITOLS model, this structure is indicative of a replication bubble with an RNA patch on the parental H-strand (Fig. 5 B, upper panel, and C). In agreement with this notion, removal of RNA by RNase A and RNase H treatment abolished the bubble arc (Fig. 5 B, middle panel).

Our 2D-AGE analysis was thus in agreement with previous reports supporting the RITOLS model. However, we hypothesized that the bubble was not a *bona fide* replication product, but instead is created during mtDNA preparation. The presence of excess amounts of mature mitochondrial transcripts presented a problem for the 2D-AGE analysis, since the mtDNA purification protocol included a proteinase K treatment step [29], [31]. Proteolytic removal of mtSSB would expose the parental H-strand in its single-stranded conformation and the large excess of RNA present could then hybridize to the H-strand and obscure the subsequent 2D-AGE analysis. The RNA-DNA hybrids formed in this way could resemble the replications products seen as evidence for the RITOLS model. To address this possibility, we investigated if it was possible to recreate the bubble arc *in vitro* by simply mixing purified mtDNA (RNase A and Rnase H treated) with purified, processed transcripts (DNase I treated) (mixing the samples depicted in Fig. 5 A, lanes 3 and 4). As demonstrated in Fig. 5B, lower panel, the mixing reaction recreated a bubble arc, which was clearly visible in 2D-AGE analysis. Our analysis therefore demonstrated that a simple reannealing reaction during the preparation of mtDNA can explain the observed replication bubble intermediate seen as evidence for the RITOLS model.

## Discussion

The SDM of mtDNA replication was proposed already in 1972 based on density gradient ultracentrifugation and electron microscopy studies of replicative intermediates [32]. Later biochemical characterization have verified the principles of this model and explained the molecular mechanisms of the individual steps [6], [17], [33]. However, SDM is still not generally accepted and proponents of alternative models, RITOLS and strand-coupled DNA replication, have pointed to the lack of sufficient *in vivo* data supporting SDM [21], [22].

The mtSSB protein is essential for mtDNA maintenance, but its functional role *in vivo* is not completely understood [27], [34], [35]. Others have demonstrated that mtSSB is actively recruited to nucleoids during mtDNA replication *in vivo* [34]. In the present work, we have used ChIP to analyze mtSSB distribution *in vivo*. In agreement with early electron microscopy studies of rat mtDNA, our data demonstrate that mtSSB covers displaced single strands of replicative intermediates [36]. With the exception of the triple-stranded D-loop region, we find that mtSSB associates exclusively with the H-strand. The protein is not evenly distributed over the genome. Instead, we observe high levels of mtSSB just downstream of the D-loop region, at the 3′-end of 7S DNA, which are followed by a gradual decrease of mtSSB occupancy towards OriL. A second, small peak of mtSSB occurs just after OriL, but it fades away as towards the D-loop region. The mtSSB profile is therefore in excellent agreement with the SDM. According to this model, the parental H-strand close to the D-loop region will remain single-stranded for a much longer time than regions closer to OriL, explaining the higher levels of mtSSB. Similarly, the region just after OriL will remain single stranded for a longer time than regions closer to OriH (Fig. 4C, upper panel). Our findings strongly argue against the RITOLS mode of mtDNA replication. According to this model, we would not expect to find mtSSB on the parental H-strand, since processed RNA species should associate with this strand, leaving no space for mtSSB to bind (Fig. 4 C, lower panel) [22]. Our data also argue against strand-coupled replication, which would predict that mtSSB is evenly distributed over the mtDNA molecule. The lagging strand is synthesized in short pieces (Okazaki fragment) and mtSSB should therefore remain associated with each fragment for about the same length of time (Fig. 4 C, middle panel).

Our findings demonstrate that mtSSB helps to restrict initiation of lagging strand DNA synthesis to OriL. POLRMT utilizes ATP as the priming nucleotide and can initiate primer synthesis from single-stranded DNA that contains at least one T-residue. Given its low sequence specificity, POLRMT could potentially initiate primer synthesis at multiple sites on the displaced parental H-strand. The mtSSB may not only stabilize the parental H-strand in its single-stranded conformation, but also prevent random priming events and unregulated initiation of L-strand synthesis. The mtSSB protein is abundant (2,100 mtSSB per mtDNA) and thus present in sufficient quantities to completely cover the parental H-strand and block unspecific primer synthesis during mtDNA replication (Figs. 1B and 2A). An interesting possibility that we will address in the future is that mtSSB may not only act to restrict POLRMT synthesis at non-specific DNA sequences, but also actively recruits POLRMT to OriL in order to initiate L-strand mtDNA replication.

Bubble type replication intermediates with associated RNA have been used as an *in vivo* proof for RITOLS. As demonstrated here, these intermediates can be recreated by a simple annealing reaction *in vitro*. During purification of mtDNA, mtSSB is removed by proteinase K treatment, exposing the parental H-strand in its single-stranded conformation. Under these conditions, the vast excess of mitochondrial transcripts present in mtDNA preparations may anneal to their complementary regions in the parental, single-stranded H-strand and form structures, which can easily be misinterpreted as replications intermediates. Strictly taken, our 2D-AGE experiments do not exclude the presence of RNA on the lagging strand *in vivo*, but we clearly demonstrate that the methods used in previous studies [21], [28], [31] can readily lead to hybridization of matured transcripts during DNA isolation due to the removal of mtSSB by proteinase K treatment. Combined with our mtSSB *in vivo* occupancy data, our findings strongly argue against the presence of the proposed RITOLS mode of replication in human cells.

To demonstrate the *in vivo* relevance of RITOLS, others have cross-linked RNA to DNA before cell lysis and isolation of mtDNA [21]. The rationale behind this approach is that if RNA forms a hybrid with DNA *in vivo*, cross-linking should protect it from digestion by RNase H. In contrast, if the RITOLS-type intermediates are formed after cell lysis and crosslinking, they should remain RNase H sensitive. Indeed, the authors of this previous study could observe a small difference in RNase H sensitivity for some of the replication intermediates. However, the identification of hybridized, crosslinked RNA molecules does not provide evidence for them playing a role in mtDNA replication, but could simply be due to ongoing transcription. Transcript processing in mitochondria is limited to 3′-end cleavage and polyadenylation, both co-transcriptional events that may take place even if some part of the transcript is crosslinked to the DNA template.

In agreement with published data from other groups, our 2D-AGE analysis of the OriH region revealed a Y-arc [19], [30]. This 2D-AGE structure corresponds to a double-stranded fork structure, which for example is observed during coupled leading- and lagging-strand DNA synthesis. This observation led to the suggestion that mtDNA replication is performed by a strand-coupled mechanism. As noted above, our mtSSB binding data does not support the existence of the SC mode of mtDNA replication *in vivo*. Could there be alternative explanation for the Y-arc at OriH? Clayton and colleagues have suggested a slightly modified version of the SDM of replication, according to which OriL is the major initiation site for initiation of L-strand synthesis, but other minor initiation sites also exists [30]. For example, it is possible that mtSSB fails to completely protect the displaced parental H-strand. Since the only requirement for POLRMT activity is the presence of a single dT, this could lead to initiation of lagging-strand DNA synthesis from sites outside OriL and the creation of a Y-arc structure in the OriH region. Another possible explanation is that the DNA replication rate might differ between H-strand and L-strand synthesis. Whereas H-strand DNA replication uses a double-stranded DNA template and relies on TWINKLE, which is a relatively slow DNA helicase, L-strand DNA replication takes place on a single-stranded DNA template and in this case TWINKLE is not required. L-strand DNA synthesis initiated from OriL may therefore reach the control region well before H-strand DNA synthesis is completed. Since OriH has been suggested to act as a pausing element for mtDNA replication, an asymmetry in replication speed will lead to the formation of a double-stranded fork structure and appearance of a Y-arc in 2D-AGE analysis. In agreement with this notion, we have previously reported that TWINKLE is a relatively slow helicase and rate limiting for DNA replication fork progression *in vitro* [25]. Future work may address how the mitochondrial replication machinery and its speed vary between the two strands *in vivo.*

## Materials and Methods

### Recombinant proteins

Human TWINKLE, mtSSB, POLγA, POLγB, POLRMT, and TFAM were expressed and purified as described previously [16], [25], [37].

### *In vitro* priming on ssDNA oligonucleotides

The reaction mixture contained 100 fmol ssDNA oligonucleotide, 10 mM Tris-HCl [pH 8.0], 25 mM MgCl_2_, 1 mM DTT, 100 µg/ml BSA, 400 µM ATP, 150 µM CTP, 10 µM GTP, 150 µM UTP, (α-32P) 2 µci GTP, 4 units RNase inhibitor (Amersham Biosciences), 500 fmol of POLRMT and 1 pmol of mtSSB (if added). After 30 min incubation at 32 °C, 12 units of RNase free DNase I (Qiagen) was added. Reactions were processed as previously described for *in vitro* transcription reactions [37] and analyzed on a 25% polyacrylamide gel containing 3 M urea in 1× TBE. The primers used were 0-dT, 1-dT, 2-dT, 3-dT, 4-dT, 5-dT, and 6-dT. The primer sequences are listed in Table S1.

### Rolling-circle mtDNA replication

For rolling-circle template formation, we cloned a DNA fragment corresponding to nts 5275–6203 of the mitochondrial human genome between the HindIII and EcoRI sites in the pBluescript SK (+) vector (Agilent Technologies; La Jolla, CA). The pBluescript SK (+) OriL construct was used as a template in site-directed PCR mutagenesis reactions to generate the mutant variant OriL-del. Constructs were confirmed by sequencing and used for ssDNA isolation following the manufacturer's protocol (Stratagene). To produce the rolling-circle DNA replication template, we annealed a 70-mer oligonucleotide (20 pmol) (5′-42[T]-ATC TCA GCG ATC TGT CTA TTT CGT TCA T-3′) to the pBluescript SK (+) OriL ssDNA (2 pmol) and the second strand was synthesized in a PCR reaction using KOD Hot Start DNA polymerase (Novagen). The samples were purified using the QIAquick PCR Purification Kit (QIAGEN). Reactions were carried out as described previously [6]. The reaction mixtures (20 µl) contained 10 fmol of indicated dsDNA template, TWINKLE (100 fmol), POL**γ**A (250 fmol), POLγB (375 fmol, calculated as dimer), POLRMT (250 fmol), and increasing amount of mtSSB as indicated in the figure legends.

### Southern blot analysis

Rolling circle reactions and Southern blot analysis was carried out as described previously [6]. For detection we used a mixture of 50-nts long radioactively probes that detected various regions in the H-strand (oligonucleotides H1, H2, H-out, H-out1, H-out2, H-out3, H-out4, H-out5, and H-out6) or L-strand (oligonucleotides H1, H2, H-out, H-out1, H-out2, H-out3, H-out4, H-out5, and H-out6). The sequences of these oligonucleotides are listed in the Table S1.

### Electrophoretic mobility shift assay (EMSA)

The OriL stem-loop conformation was analyzed with a DNA binding assay on ssDNA oligonucleotides with increasing concentrations of mtSSB (0, 5, 10, 25, 50, 100 fmol). Reactions (15 µl) contained 20 mM Tris-HCl [pH 8.0], 10 mM MgCl_2_, 100 µg/ml BSA, 1 mM DTT, 2 mM ATP, 10% Glycerol and 25 fmol of the indicated 5′-end [γ-32P] labeled ssDNA oligo. Reactions were incubated for 15 min at room temperature before separation on a 10% polyacrylamide native gel in 1 × TBE for 50 min at 150 V. The oligonucleotides used in the experiments were 6-dT, OriL-WT, and OriL+6. The sequences of these oligonucleotides are listed in Table S1.

### Chromatin immunoprecipitation and analysis

Mitochondria were isolated from HeLa cells as described in [28]. The isolated mitochondria were washed once with ice-cold PBS, and incubated in 1% formaldehyde in PBS for 10 min at room temperature. The crosslinking reaction was quenched by addition of glycine to a final concentration of 125 mM, followed by incubation for 5 additional minutes. After washing twice in ice-cold PBS, mitochondria were lysed in 25 mM HEPES-KOH (pH 7.6), 10% glycerol, 5 mM MgCl_2_, 0.5 mM EDTA, 0.5% tween-20, 0.15 M KCl, 1 mM phenylmethlsulfonylfluoride, 2 mM pepstatin A, 0.6 mM leupeptin, and 2 mM benzamidine. The mitochondrial lysates were sonicated in a Bioruptor UCD 200TM (Diagenode) for 3×10 min at high output, with intervals of 30 seconds on and 30 seconds off, and centrifuged for 5 min at 14,000×g. An aliquot of the supernatant (50 µl) was taken out as input control, whereas 400 µl were incubated with either 20 µl of human TFAM polyclonal antibody (Agrisera), 20 µl of human mtSSB polyclonal antibody (12212-1-AP, Proteintech), or 5.5 µl of rabbit IgG (ab37415, Abcam) overnight on a rotator at 4°C. Fifty µl of protein A beads (GE Healthcare) were added to the samples and incubated for 1 hour at 4°C. After wash and elution, eluted DNA samples were incubated overnight at 65°C to reverse crosslinking. RNA contamination was removed by incubation with 100 ng/ml RNaseA for 15 min at 37°C. Proteins were removed by addition of 20 µg proteinase K and incubation for 2 hrs at 56°C. DNA was purified by phenol/chloroform extraction, and ethanol precipitation. The purified DNA was used for strand-specific PCR analysis or sequencing (see below). Three independent biological replicates were carried out for ChIP analysis. Average and standard deviation were calculated and plotted in Microsoft Excel.

### ChIP analysis by strand-specific qPCR and sequencing

Strand-specific qPCR was used to monitor the levels of H- and L-strand in the ChIP material. Three primers were used in each reaction: a Tagging primer, a Tag primer, and a Reverse primer for each region analyzed. The primers used and their sequences are listed in Table S1. Primers with “H” were used for amplification of the heavy strand sequence, while primers with “L” for light strand. Reactions were initiated by annealing of the Tagging primer, which contains 11–13 nts of locus-specific sequence at its 3′-end and a 19-nts Tag sequence in its 5′-end. During the first cycle the annealed Tagging primer was extended to the end of the single-strand DNA. In subsequent cycles the generated hybrid sequence was amplified by qPCR with the Tag and Reverse primers. The reaction contains 400 nM of Tag primer, 400 nM of Reverse primer, and 8 nM Tagging primer in a reaction volume of 25 µl. The PCR cycles were as follows: 95°C for 3 min; 40°C for 5 min; increase to 72°C at a rate of 2°C/min; 94°C for 4 min; 94°C for 15 s and 64°C for 1 min (40 cycles). Quantification was performed using real time PCR Software (Bio-Rad) and Excel (Microsoft); ratios of IP/input are depicted in the figures after subtracting ratios obtained from rabbit IgG control. Standard curve was produced by different dilutions of Input, the quantity of ssDNA is calculated relative to the standards [38].

For strand-specific ChIP sequencing, the DNA was further prepared using standard protocols provided by Illumina and deep-sequenced by using Illumina's Solexa sequencer (Beijing Genomics Institute). Quality control statistics were generated with FastQC (http://www.bioinformatics.bbsrc.ac.uk/projects/fastqc).

### Quantitative immunoblot analysis of mtSSB levels

The concentration of recombinant and human mtSSB were determined by OD_280_ and Bradford measurements with Bovine Serum Albumin (Sigma-Aldrich) as standard. HeLa cell numbers were determined by a Vi-CELL XR system (Beckman-Coulter). For PAGE analyses with Criterion Tris-HCl Gels (Bio-Rad), HeLa cell pellets were resuspended in a buffer containing 100 mM Tris-Cl, pH 6.8, 4% SDS, 20% glycerol (Sigma-Aldrich) and Complete Protease Inhibitor Mixture (Roche Diagnostics), vortexed, incubated at 95°C for 5 min, and sonicated for 5 cycles at 30 s. Before PAGE, 200 mM DTT and 0.2% bromophenol blue (Sigma-Aldrich) were added. Western Blot analyses were performed with polyclonal antibodies against human mtSSB (Proteintech). Anti-rabbit IgG horseradish peroxidase linked secondary antibodies were visualized by Amersham ECL Western Blotting Detection Reagents (GE Healthcare). The quantification of mtSSB was performed by interpolating the intensity of the bands in the immunoblot in a calibration curve made with known amounts of recombinant proteins using the Quantity One 4.6 software (Bio-Rad).

### Quantification of mtDNA content

HeLa cell numbers were determined as described above and lysed in QuantiLyse buffer and quantified as described previously [39].

### 2D-AGE analysis

HeLa cells were routinely grown in Dulbecco's modified Eagle's medium (DMEM) (Life Technologies) with 10% fetal bovine serum. After centrifugation, cell pellets were resuspended in nine volumes of homogenization buffer (225 mM Mannitol, 75 mM sucrose, 10 mM Hepes-NaOH [pH 7.4], 10 mM EDTA) containing 1 mg/ml BSA and 1 mM DTT, and cells were broken in a 7-ml Wheaton homogenizer with a tight-fitting glass pestle. Mitochondria were purified and lysed, protease treated and as described previously [29]. Mitochondrial nucleic acid was precipitated with isopropanol and stored in aliquots at −20°C. When indicated purified mtDNA (15 ug) was further treated with 15 U of RNase H and 50 U of RNase A for 1 hr at 37°C or with 50 U DNase I for 1 hr at 4°C.

HincII digestion was performed under conditions recommended by the manufacturer (New England Biolabs) for 3 hrs using 2 µg mtDNA. Neutral 2D-AGE was performed following published protocols [40], [41]. Southern blot analysis of 2D gels were hybridized to a specific region of human mtDNA (mtDNA 13636-1006) by 2 hrs incubation at 65°C in hybridizing buffer (Amersham rapid-hyb buffer) under conditions recommended by the manufacturer (GE Healthcare). Posthybridization washes were 2× SSC, 0.1% SDS, twice for 10 min followed by 0.2× SSC, 0.1% SDS, twice for 15 min at 65°C. Filters were exposed to X-ray film. Radiolabeled probe against the region (mtDNA 13636-1006) was generated using the Prime-it II kit (Agilent Tech) and a gel purified PCR fragment (spanning a region of 14641-15590 in mtDNA) as a template.

### Northern blotting

Purified mtDNA treated with DNase I (Fig. 5A, lane 3) was separated on a 1% formaldehyde agarose gel. The RNA was transferred onto a nylon Hybond-N+ membrane (GE Healthcare) using capillary blotting with 10× SSC buffer with 0.5× TBE buffer. After transfer of the RNA, the membrane was cross-linked using UV radiation. Strand specific DNA oligonucleotides against ND4, CYTB and COX2 were radioactively labeled and then hybridized to the membrane.

### Accession numbers

Data have been deposited in the NCBI Sequence Read Archive (SRA) under accession number SRX481175.

## Supporting Information

Figure S1 MtSSB abolishes primer synthesis on a linear template, but not at OriL. Primase assays were performed on stem-loop forming oligonucleotides corresponding to WT OriL (lanes 1 and 3) or a linear derivative lacking the double-stranded stem (lanes 2 and 4). MtSSB (1 pmol) was added when indicated.
(EPS)

Figure S2 Processed RNA transcripts are co-purified with DNA. Northern blot analysis as described in Materials and Methods of purified mtDNA treated with DNase I (visualized in Fig. 5 A, lane 3) with probes against ND4, CYTB and COX3.
(EPS)

Table S1 A list of oligonucleotides used in this study.
(DOC)
