# Supplementary material for: In Vivo Occupancy of Mitochondrial Single-Stranded DNA Binding Protein Supports the Strand Displacement Mode of DNA Replication
Source: PLoS Genet. 2014 Dec 4;10(12):e1004832. doi: 10.1371/journal.pgen.1004832 (PMC4256270; doi:10.1371/journal.pgen.1004832)
Supplement: Table S1 — A list of oligonucleotides used in this study. (DOC) [file pgen.1004832.s003.doc]

**Sequences of oligonucleotides used in this study.**

| **Name** | **Sequence (5′ to 3′ direction)** | **Comments** |
| --- | --- | --- |
| 0-dT | AAC ACC AGC AGA ACA AAA AAA ACA AGC AAC ACA CAG AAG ACA | Used for primase assay |
| 1-dT | AAC ACC AGC AGA ACT AAA AAA ACA AGC AAC ACA CAG AAG ACA | Used for primase assay |
| 2-dT | AAC ACC AGC AGA ACT TAA AAA ACA AGC AAC ACA CAG AAG ACA | Used for primase assay |
| 3-dT | AAC ACC AGC AGA ACT TTA AAA ACA AGC AAC ACA CAG AAG ACA | Used for primase assay |
| 4-dT | AAC ACC AGC AGA ACT TTT AAA ACA AGC AAC ACA CAG AAG ACA | Used for primase assay |
| 5-dT | AAC ACC AGC AGA ACT TTT TAA ACA AGC AAC ACA CAG AAG ACA | Used for primase assay |
| 6-dT | AAC ACC AGC AGA ACT TTT TTA ACA AGC AAC ACA CAG AAG ACA | Used for primase assay and EMSA |
| H1 | TTA TTC GGC GCA TGA GCT GGA GTC CTA GGC ACA GCT CTA AGC CTC CTT AT | Used for detection of H-strand synthesis in Southern blotting |
| H2 | CCT ATC TCC CCT TTT ATA CTA ATA ATC TTA TAG AAA TTT AGG TTA AAT AC | Used for detection of H-strand synthesis in Southern blotting |
| H-out | GGT ATC ATT GCA GCA CTG GGG CCA GAT GGT AAG CCC TCC CGT ATC GTA GT | Used for detection of H-strand synthesis in Southern blotting |
| H-out1 | AAC TAC TTA CTC TAG CTT CCC GGC AAC AAT TAA TAG ACT GGA TGG AGG CG | Used for detection of H-strand synthesis in Southern blotting |
| H-out2 | ATT GAA AAA GGA AGA GTA TGA GTA TTC AAC ATT TCC GTG TCG CCC TTA TT | Used for detection of H-strand synthesis in Southern blotting |
| H-out3 | AGC GGC GCA TTA AGC GCG GCG GGT GTG GTG GTT ACG CGC AGC GTG ACC GC | Used for detection of H-strand synthesis in Southern blotting |
| H-out4 | AGC GGG CAG TGA GCG CAA CGC AAT TAA TGT GAG TTA GCT CAC TCA TTA GG | Used for detection of H-strand synthesis in Southern blotting |
| H-out5 | TAA GTC GTG TCT TAC CGG GTT GGA CTC AAG ACG ATA GTT ACC GGA TAA GG | Used for detection of H-strand synthesis in Southern blotting |
| H-out6 | AGG TGC CTC ACT GAT TAA GCA TTG GTA ACT GTC AGA CCA AGT TTA CTC AT | Used for detection of H-strand synthesis in Southern blotting |
| L1 | ATA AGG AGG CTT AGA GCT GTG CCT AGG ACT CCA GCT CAT GCG CCG AAT AA | Used for detection of L-strand synthesis in Southern blotting |
| L2 | GTA TTT AAC CTA AAT TTC TAT AAG ATT ATT AGT ATA AAA GGG GAG ATA GG | Used for detection of L-strand synthesis in Southern blotting |
| L-out | ACT ACG ATA CGG GAG GGC TTA CCA TCT GGC CCC AGT GCT GCA ATG ATA CC | Used for detection of L-strand synthesis in Southern blotting |
| L-out1 | CGC CTC CAT CCA GTC TAT TAA TTG TTG CCG GGA AGC TAG AGT AAG TAG TT | Used for detection of L-strand synthesis in Southern blotting |
| L-out2 | AAT AAG GGC GAC ACG GAA ATG TTG AAT ACT CAT ACT CTT CCT TTT TCA AT | Used for detection of L-strand synthesis in Southern blotting |
| L-out3 | GCG GTC ACG CTG CGC GTA ACC ACC ACA CCC GCC GCG CTT AAT GCG CCG CT | Used for detection of L-strand synthesis in Southern blotting |
| L-out4 | CCT AAT GAG GAG CTA ACT CAC ATT AAT TGC GTT GCG CTC ACT GCC CGC T | Used for detection of L-strand synthesis in Southern blotting |
| L-out5 | CCT TAT CCG GTA ACT ATC GTC TTG AGT CCA ACC CGG TAA GAC ACG ACT TA | Used for detection of L-strand synthesis in Southern blotting |
| L-out6 | ATG AGT AAA CTT GGT CTG ACA GTT ACC AAT GCT TAA TCA GTG AGG CAC CT | Used for detection of L-strand synthesis in Southern blotting |
| OriL-WT | GGG CTT CTC CCG CCT TTT TTC CCG GCG GCG GGA GAA GTA GAT TGA AG | Used for EMSA |
| OriL+6 | GGG CTT CTC CCG CCC CCG CCT TTT TTC CCG GCG GCG GGG GCG GGA GAA GTA GAT TG | Used for EMSA |
| CYTB-Tagging-H | AAG GAG CGC AGC GCC TGT ATG C ctg atc ctc | Used for strand-specific qPCR analysis |
| CYTB-Tag-H | AAG GAG CGC AGC GCC TGT ATG C | Used for strand-specific qPCR analysis |
| CYTB-Reverse-H | GGC GTG AAG GTA GCG GAT GAT TCA GCC | Used for strand-specific qPCR analysis |
| CYTB-Tagging-L | AAG GAG CGC AGC GCC TGT AGT Gaa ggt agc g | Used for strand-specific qPCR analysis |
| CYTB-Tag-L | AAG GAG CGC AGC GCC TGT AGT G | Used for strand-specific qPCR analysis |
| CYTB-Reverse-L | GCC TGC CTG ATC CTC CAA ATC ACC ACA | Used for strand-specific qPCR analysis |
| ND5-Tagging-H | AAG GAG CGC AGC GCC TGT AAG CAG CAG CAG G | Used for strand-specific qPCR analysis |
| ND5-Tag-H | AAG GAG CGC AGC GCC TGT AAG C | Used for strand-specific qPCR analysis |
| ND5-Reverse-H | GCT AGG GGG TGG AAG CGG ATG AGT AAG A | Used for strand-specific qPCR analysis |
| ND5-Tagging-L | AAG GAG CGC AGC GCC TGT AGT GGA AGC GGA | Used for strand-specific qPCR analysis |
| ND5-Tag-L | AAG GAG CGC AGC GCC TGT AGT G | Used for strand-specific qPCR analysis |
| ND5-Reverse-L | CTA GCA GCA GCA GGC AAA TCA GCC C | Used for strand-specific qPCR analysis |
| ND4-Tagging-H | AAG GAG CGC AGC GCC TGT ACA CTC TCA CTG C | Used for strand-specific qPCR analysis |
| ND4-Tag-H | AAG GAG CGC AGC GCC TGT ACA C | Used for strand-specific qPCR analysis |
| ND4-Reverse-H | GGG GGC TTC GAC ATG GGC TTT AGG | Used for strand-specific qPCR analysis |
| ND4-Tagging-L | AAG GAG CGC AGC GCC TGT ATC GAC ATG GGC T | Used for strand-specific qPCR analysis |
| ND4-Tag-L | AAG GAG CGC AGC GCC TGT ATC G | Used for strand-specific qPCR analysis |
| ND4-Reverse-L | GGC TCC CTT CCC CTA CTC ATC GCA C | Used for strand-specific qPCR analysis |
| COX3-Tagging-H | AAG GAG CGC AGC GCC TGT ACA CTC CAT AAC GC | Used for strand-specific qPCR analysis |
| COX3-Tag-H | AAG GAG CGC AGC GCC TGT ACA C | Used for strand-specific qPCR analysis |
| COX3-Reverse-H | GGT GTG TGG TGG CCT TGG TAT GTG C | Used for strand-specific qPCR analysis |
| COX3-Tagging-L | AAG GAG CGC AGC GCC TGT ACT TGG TAT GTG CT | Used for strand-specific qPCR analysis |
| COX3-Tag-L | AAG GAG CGC AGC GCC TGT ACT T | Used for strand-specific qPCR analysis |
| COX3-Reverse-L | GCC CTC CTA ATG ACC TCC GGC C | Used for strand-specific qPCR analysis |
| COX1-Tagging-H | AAG GAG CGC AGC GCC TGT AAG CAG GTG TCT C | Used for strand-specific qPCR analysis |
| COX1-Tag-H | AAG GAG CGC AGC GCC TGT AAG C | Used for strand-specific qPCR analysis |
| COX1-Reverse-H | GAT GCC AGC AGC TAG GAC TGG GAG AG | Used for strand-specific qPCR analysis |
| COX1-Tagging-L | AAG GAG CGC AGC GCC TGT AAG GAC TGC TGT G | Used for strand-specific qPCR analysis |
| COX1-Tag-L | AAG GAG CGC AGC GCC TGT AAG G | Used for strand-specific qPCR analysis |
| COX1-Reverse-L | GCA GGT GTC TCC TCT ATC TTA GGG GCC | Used for strand-specific qPCR analysis |
| ND2-Tagging-H | AAG GAG CGC AGC GCC TGT ACT GAC ATC CGG | Used for strand-specific qPCR analysis |
| ND2-Tag-H | AAG GAG CGC AGC GCC TGT ACT G | Used for strand-specific qPCR analysis |
| ND2-Reverse-H | GCG TAG CTG GGT TTG GTT TAA TCC ACC TC | Used for strand-specific qPCR analysis |
| ND2-Tagging-L | AAG GAG CGC AGC GCC TGT ATC CAC CTC AAC T | Used for strand-specific qPCR analysis |
| ND2-Tag-L | AAG GAG CGC AGC GCC TGT ATC C | Used for strand-specific qPCR analysis |
| ND2-Reverse-L | CCT CTG ACA TCC GGC CTG CTT CTT CTC | Used for strand-specific qPCR analysis |
| ND1-Tagging-H | AAG GAG CGC AGC GCC TGT AGA GCA GTA GCC C | Used for strand-specific qPCR analysis |
| ND1-Tag-H | AAG GAG CGC AGC GCC TGT AGA G | Used for strand-specific qPCR analysis |
| ND1-Reverse-H | GGC CAA GGG TCA TGA TGG CAG GAG | Used for strand-specific qPCR analysis |
| ND1-Tagging-L | AAG GAG CGC AGC GCC TGT AGT CAT GAT GGC A | Used for strand-specific qPCR analysis |
| ND1-Tag-L | AAG GAG CGC AGC GCC TGT AGT C | Used for strand-specific qPCR analysis |
| ND1-Reverse-L | GCA CTG CGA GCA GTA GCC CAA ACA A | Used for strand-specific qPCR analysis |
| RNR2-Tagging-H | AAG GAG CGC AGC GCC TGT AAC CGT GCA AAG G | Used for strand-specific qPCR analysis |
| RNR2-Tag-H | AAG GAG CGC AGC GCC TGT AAC C | Used for strand-specific qPCR analysis |
| RNR2-Reverse-H | CCG CCT CTT CAC GGG CAG GTC | Used for strand-specific qPCR analysis |
| RNR2-Tagging-L | AAG GAG CGC AGC GCC TGT AGT GTT ATG CCC G | Used for strand-specific qPCR analysis |
| RNR2-Tag-L | AAG GAG CGC AGC GCC TGT AGT G | Used for strand-specific qPCR analysis |
| RNR2-Reverse-L | GCG GTA CCC TAA CCG TGC AAA GGT | Used for strand-specific qPCR analysis |
